# Supplementary material for: Neuropixels reveal structure-function relationships in monkey V1 in vivo
Source: bioRxiv. 2025 Jun 25:2025.05.14.653875. Originally published 2025 May 18. Preprint. [Version 2] doi: 10.1101/2025.05.14.653875 (PMC12132580; doi:10.1101/2025.05.14.653875)
Supplement: 1 [file NIHPP2025.05.14.653875V2-supplement-1.pdf]

bioRxiv preprint doi: https://doi.org/10.1101/2025.05.14.653875; this version posted June 25, 2025. The copyright holder for this preprint (which was not certified by peer review) is the author/funder, who has granted bioRxiv a license to display the preprint in perpetuity. It is made available under aCC-BY 4.0 International license.

Supplemental materials

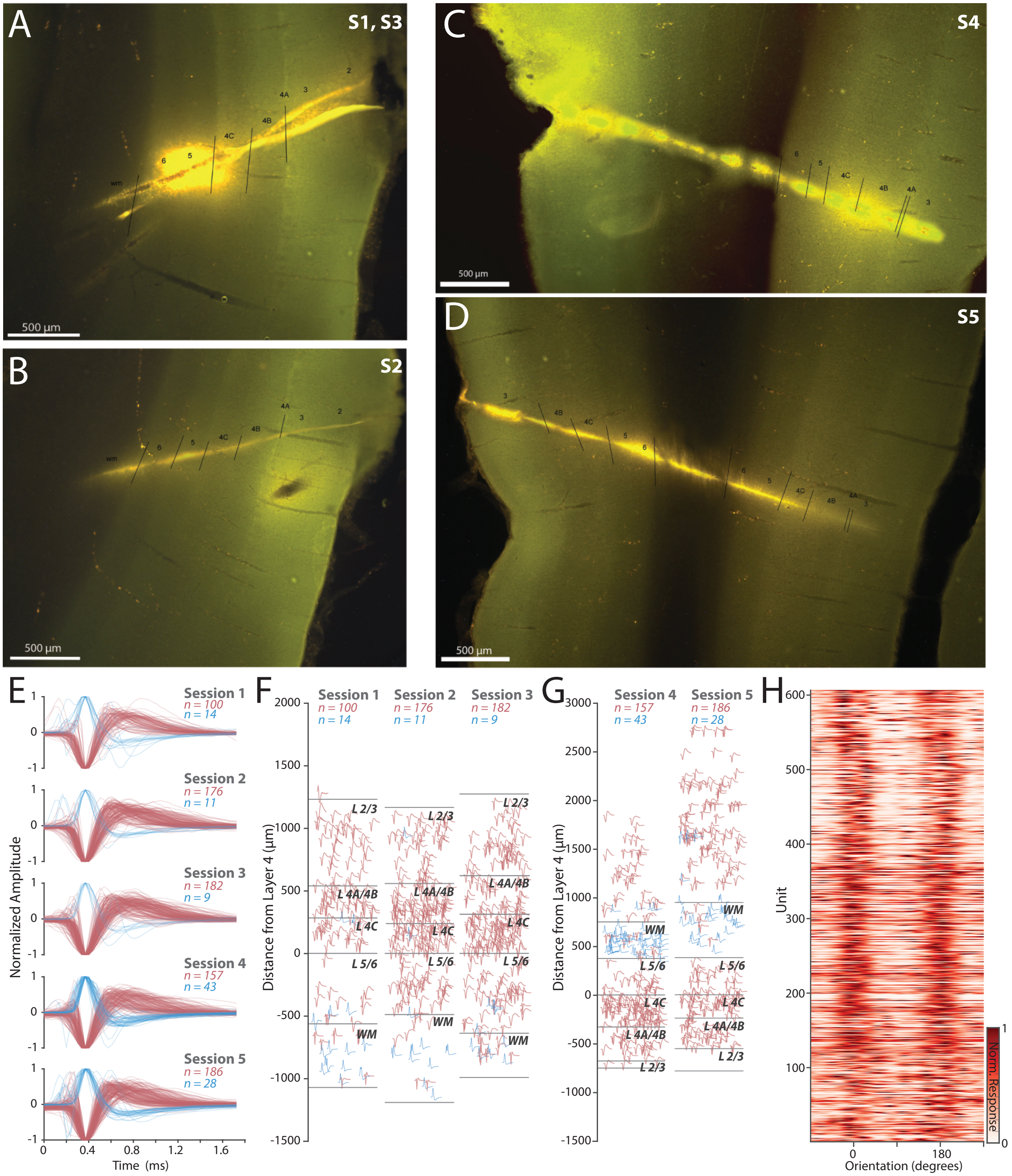

Figure S1: **Histological verification of penetrations and neuronal waveforms from each session** (**A-D**) *Left*, Dye tracks of monkey 1 (M1) for sessions 1 and 3 (A), session 2 (B). *Right*, Dye tracks of monkey 2 (M2) for session 4 (C) and session 5 (D), all with delineations of laminar boundaries. (**E**) Curated waveforms for each of the five sessions. (**F-G**) Laminar organization of waveforms in sessions 1-3 (F, M1), and waveforms in sessions 4-5 (G, M2) (**H**) The normalized response tuning aligned to overall preferred orientation for 606 visually active neurons, sorted top to bottom by depth from superficial to deep.

bioRxiv preprint doi: https://doi.org/10.1101/2025.05.14.653875; this version posted June 25, 2025. The copyright holder for this preprint (which was not certified by peer review) is the author/funder, who has granted bioRxiv a license to display the preprint in perpetuity. It is made available under aCC-BY 4.0 International license.

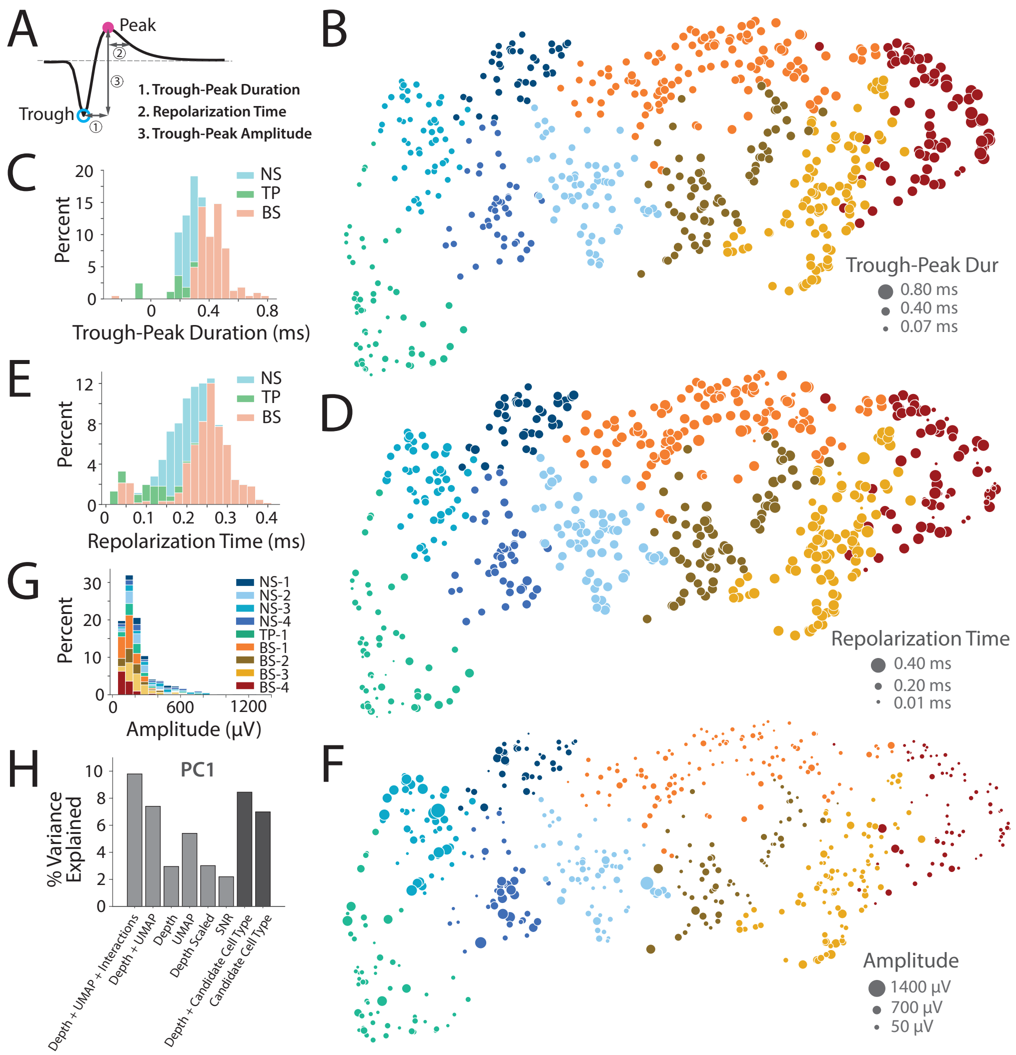

Figure S2: **WaveMAP clusters lawfully reflect classic features** (**A**) Classic features of the extracellular waveform include peak (in pink solid circle), trough (in blue open circle), trough-peak duration (T-P Dur), repolarization time from 1/2 peak (Rep Time), and normalized trough-peak amplitude (T-P Amp). (**B**) UMAP X and UMAP Y plot with unit markers sized by trough-peak duration. UMAP X is strongly correlated with T-P Dur. (**C**) Histogram of all Trough-Peak Durations showing distributions of general categories (Tri-phasic, Narrow, and Broad). Note the distribution is unimodal and not easily separated into clusters. (**D**) UMAP X and UMAP Y plot with unit markers sized by 1/2 peak repolarization time. Again note strong correlation with repolarization time. (**E**) Histogram of all 1/2 peak repolarization times showing distributions of general categories (Tri-phasic, Narrow, and Broad). Note again the unimodal distribution of these features. (**F**) UMAP X and UMAP Y plot with unit markers sized by trough-peak amplitude, a variable not included in the UMAP analysis. The results suggest that some clusters have larger amplitudes than others. (**G**) Histogram of all amplitudes showing distributions for all clusters. (**H**) We performed PCA on the functional properties, orientation index, orientation circular variance, direction index, direction circular variance, orientation tuning bandwidth, and simple complex, of all visually responsive neurons to summarize the visual functional response of the neurons. We examined if this functional visual response could be explained by various predictors of cell type using a multiple linear regression. The predictors we used are UMAP coordinates, Depth, and various interaction terms between UMAP X coordinate and depth, UMAP Y coordinate and depth, and UMAP X, Y, and depth. As a control, we measured the amount of variance explained by signal-to-noise ratio to ensure our results were not a trivial artifact of spike sorting. Overall, both UMAP X and Y, Depth and candidate cell type predicted almost 10% of the variance in visual functional responses. Notably, the candidate cell type label and depth explained comparable amounts of variance as the depth and the UMAP coordinates.

bioRxiv preprint doi: https://doi.org/10.1101/2025.05.14.653875; this version posted June 25, 2025. The copyright holder for this preprint (which was not certified by peer review) is the author/funder, who has granted bioRxiv a license to display the preprint in perpetuity. It is made available under aCC-BY 4.0 International license.

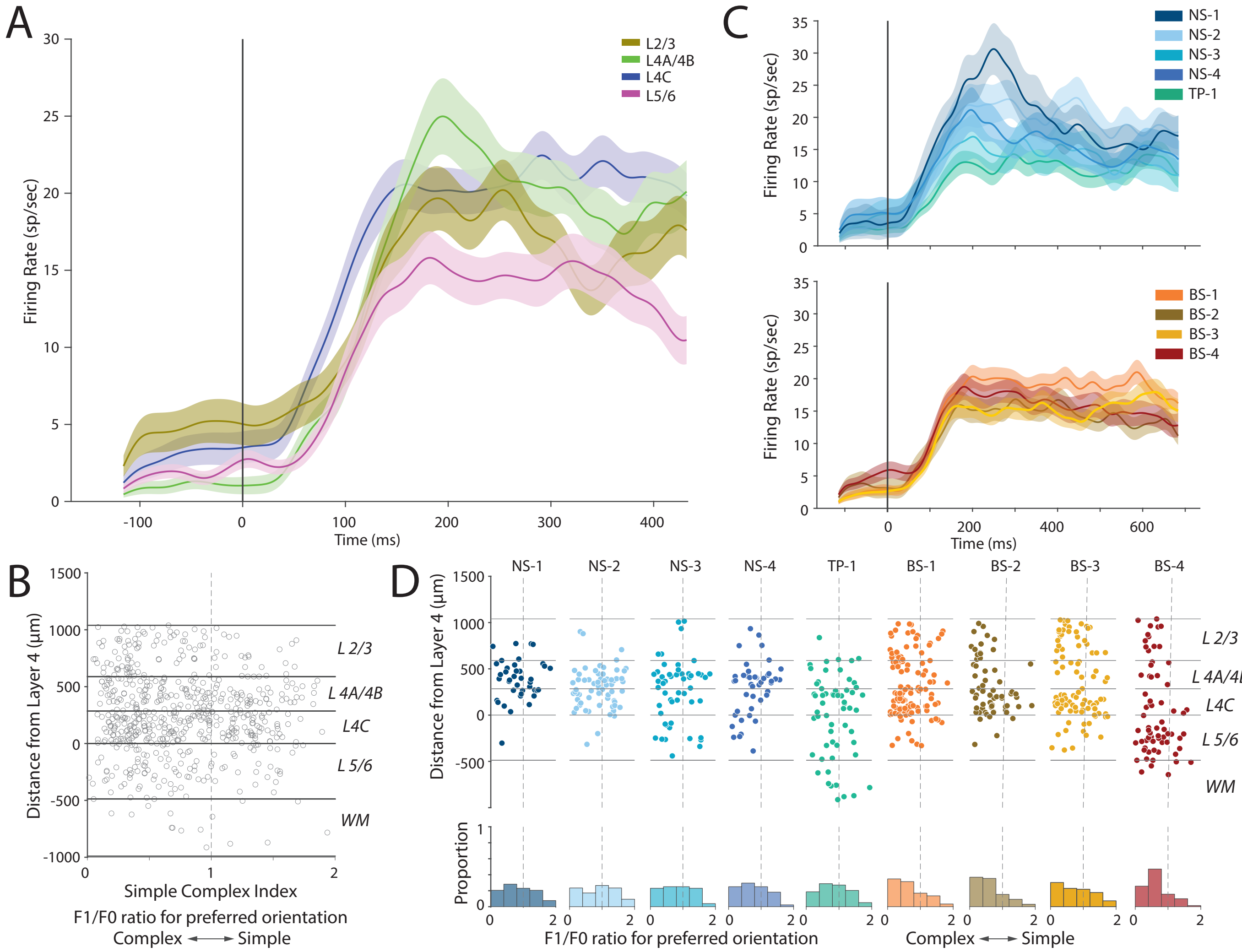

Figure S3: **Neuropixels recordings in V1 are consistent with classical studies** (**A**) Average PSTH by layer. The black line indicates the initiation of the visual stimulus. (**B**) Scatter plot of modularity index (simple-complex index) as a function of scaled laminar depth of all units. Orientation selectivity is found in all layers but strongest in layer 4 consistent with classical reports (21). (**C**) Average PSTH by cluster. The black line indicates the initiation of the visual stimulus. *Top*, Mean PSTHs of "narrow" clusters. *Bottom*, Mean PSTHs of "broad" clusters. (**D**) There showed no strong indications that simple and complex cells mapped onto any one candidate cell type, although the broad-spiking clusters generally trended towards more complex responses. *Top*, Scatter plot of modularity index (simple-complex index) and scaled laminar depth separated by cluster. *Bottom*, Panel below the scatter plots shows histograms of the simple-complex index. The vertical dashed center lines show the boundary between complex (left of center) and simple (right of center).

bioRxiv preprint doi: https://doi.org/10.1101/2025.05.14.653875; this version posted June 25, 2025. The copyright holder for this preprint (which was not certified by peer review) is the author/funder, who has granted bioRxiv a license to display the preprint in perpetuity. It is made available under aCC-BY 4.0 International license.

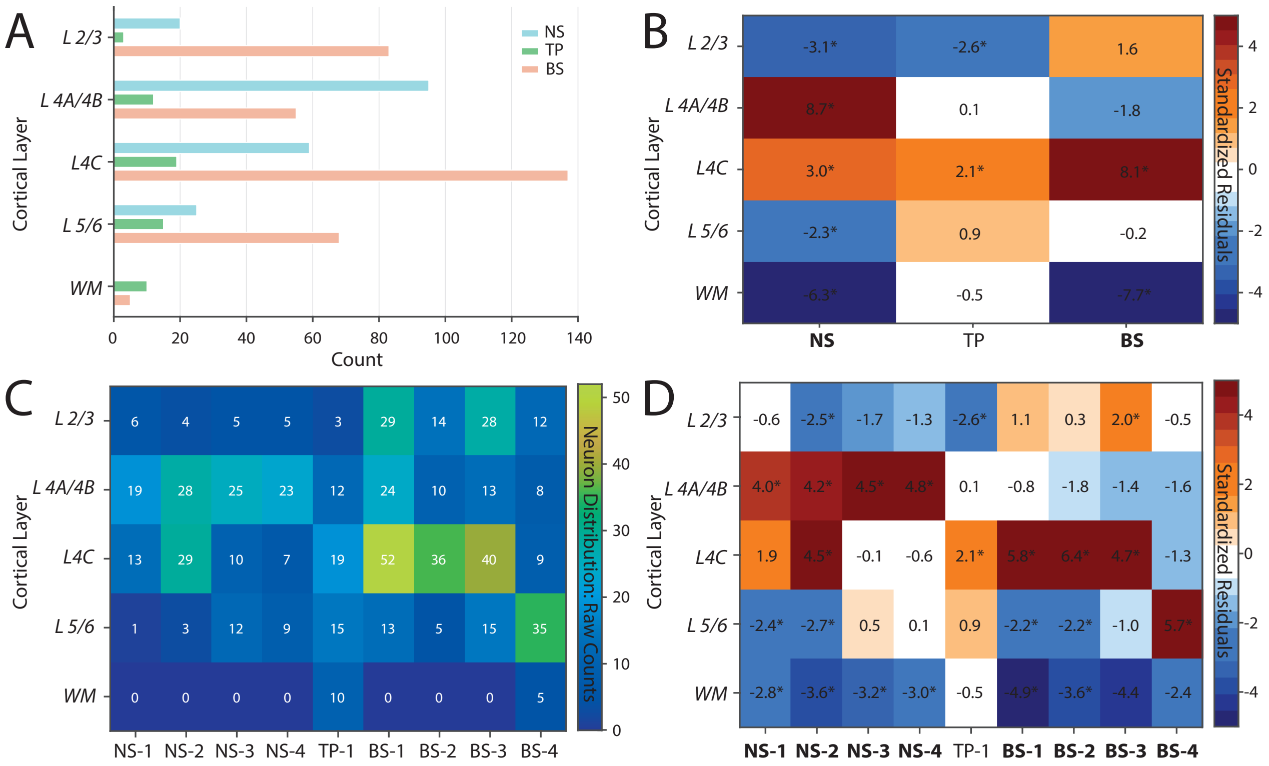

Figure S4: **Narrow-spiking groups are concentrated in Layer 4A/4B/4C** (**A**) Histogram showing neuron count per layer separated by broad categories, narrow-spiking (NS), tri-phasic (TP), and broad-spiking (BS). (**B**) Under the null hypothesis that each layer (df = 4) should have the same proportion of neurons across the three broad categories (NS, TP, and BS), we performed a chi-square analysis to compare the observed distributions. Bold text indicates the category showed significance at $p < 0.00001$, otherwise indicates significance at $p < 0.05$. The heatmap shows standardized residuals, which are the difference between the observed and expected, divided by the square root of the expected count. * indicates a value $\pm$ 2.0 which suggests significance at $p < 0.05$. A positive value indicates a greater number observed than expected, and a negative indicates a lower number observed than expected. We see that the NS category is overrepresented in Layer 4A/4B and 4C. (**C**) Raw counts of neuron distribution by layer and cluster. (**D**) Under the null hypothesis that each layer (df = 4) should have the same proportion of neurons across each cluster, we performed a chi-square analysis to compare the observed distributions. Bold text indicates the cluster showed significance at $p < 0.00001$, otherwise indicates significance at $p < 0.05$. The heatmap shows standardized residuals, which are the difference between the observed and expected, divided by the square root of the expected count. * indicates a value $\pm$ 2.0 which suggests significance at $p < 0.05$. A positive value indicates a greater number observed than expected, and a negative indicates a lower number observed than expected. We see that all NS subtypes are overrepresented in Layers 4A/4B and NS-1 and NS-2 are overrepresented in Layer 4C.

bioRxiv preprint doi: https://doi.org/10.1101/2025.05.14.653875; this version posted June 25, 2025. The copyright holder for this preprint (which was not certified by peer review) is the author/funder, who has granted bioRxiv a license to display the preprint in perpetuity. It is made available under aCC-BY 4.0 International license.

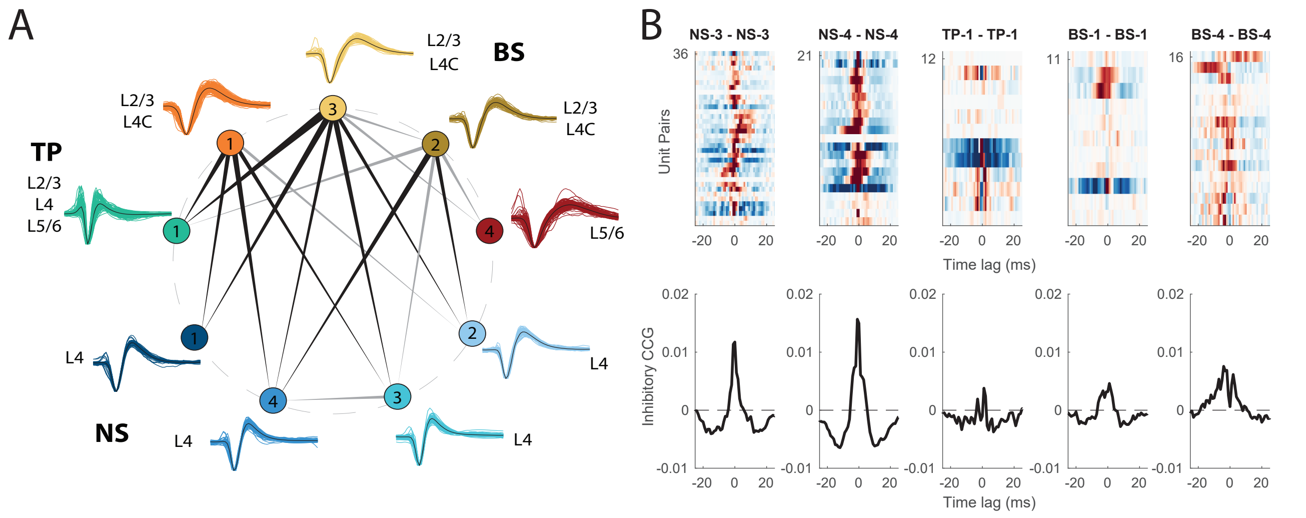

Figure S5: **Excitatory and inhibitory cross-correlation analysis shows ordered connectivity between clusters** (**A**) Summary diagram of excitatory CCGs between clusters. Line thickness indicates the directionality of the CCG, from leading (thin line) cluster to lagging (thick line) cluster, with line width weighted by median lead-lag index. Black colored lines have $p < 0.001$, grey colored lines have $p < 0.01$. Overall, narrow-spiking neurons that are strongly localized to L4 and L5/6 lead broad-spiking neurons in L2/3 and L4, perhaps due to strong inputs into layer 4 neurons. (**B**) *Top*, Inhibitory CCGs between pairs of cells within each cluster. Only clusters which contained more than 10 pairs of cells are included. NS-3 and NS-4 show more inhibitory connections within their pairs than TP-1, BS-1, and BS-4. *Bottom*, Mean inhibitory autocorrelogram trace per each cluster.

bioRxiv preprint doi: https://doi.org/10.1101/2025.05.14.653875; this version posted June 25, 2025. The copyright holder for this preprint (which was not certified by peer review) is the author/funder, who has granted bioRxiv a license to display the preprint in perpetuity. It is made available under aCC-BY 4.0 International license.

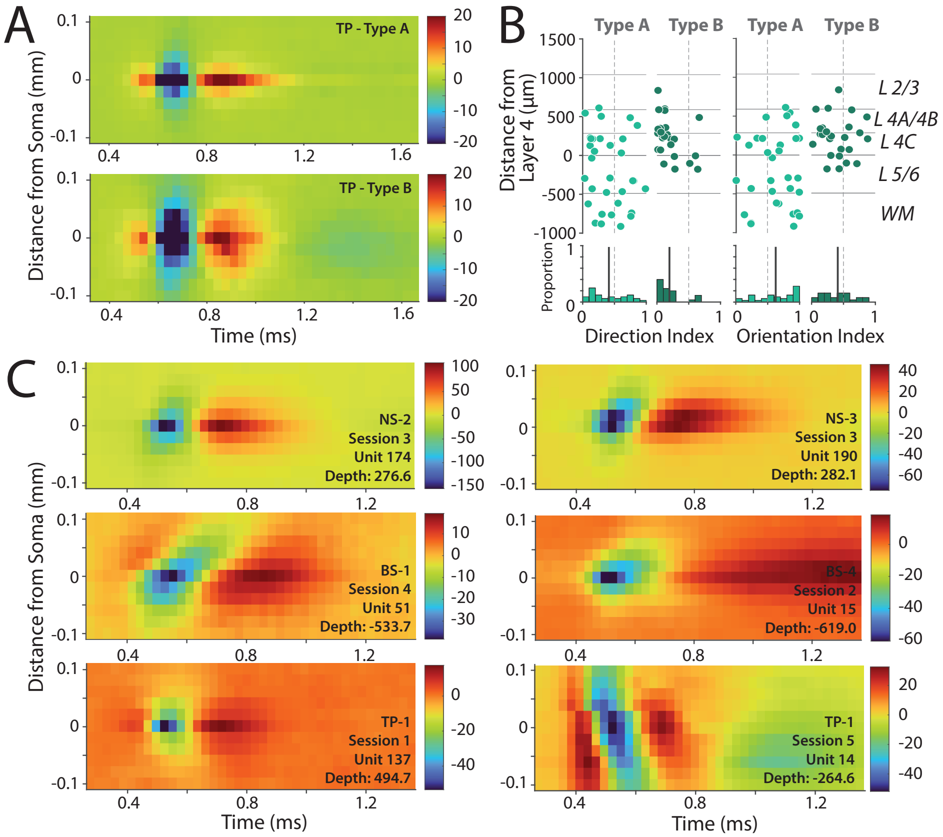

Figure S6: **Spatial profiles show more hetereogeneity than cluster features typically capture** (**A**) Average multi-channel waveforms of tri-phasic cluster Type A ($n = 32$), and Type B ($n = 25$). Type A and Type B were manually classified on the criteria of spread (number of channels on which an extracellular action potential waveform continuum was observed). Of the 59 TP-1 Waveforms that were visually responsive, 2 were identified as artifactual. (**B**) *Top*, Laminar distribution of TP-1 Type A and Type B. The x-axis shows (from left to right) direction index and orientation index. A Wilcoxon rank sum test showed that the Type B population has significantly less direction selectivity ($p < 0.01$) and orientation selectivity ($p < 0.05$) than Type A. *Bottom*, Panel below the scatter plots shows histograms of the direction and orientation selectivity index. The bold line indicates the median orientation and direction index for each cluster. The vertical dashed center lines show the boundary between not selective (left of center) and selective (right of center). (**C**) Example multichannel waveforms from various clusters and sessions. *Upper Left*, "Narrow" Cluster 2 waveform. *Lower Left*, "Narrow" Cluster 6 waveform showing backpropagation. *Upper Right*, Tri-phasic Cluster 7 waveform. *Lower Right*, "Broad" waveform showing backpropagation.

bioRxiv preprint doi: https://doi.org/10.1101/2025.05.14.653875; this version posted June 25, 2025. The copyright holder for this preprint (which was not certified by peer review) is the author/funder, who has granted bioRxiv a license to display the preprint in perpetuity. It is made available under aCC-BY 4.0 International license.

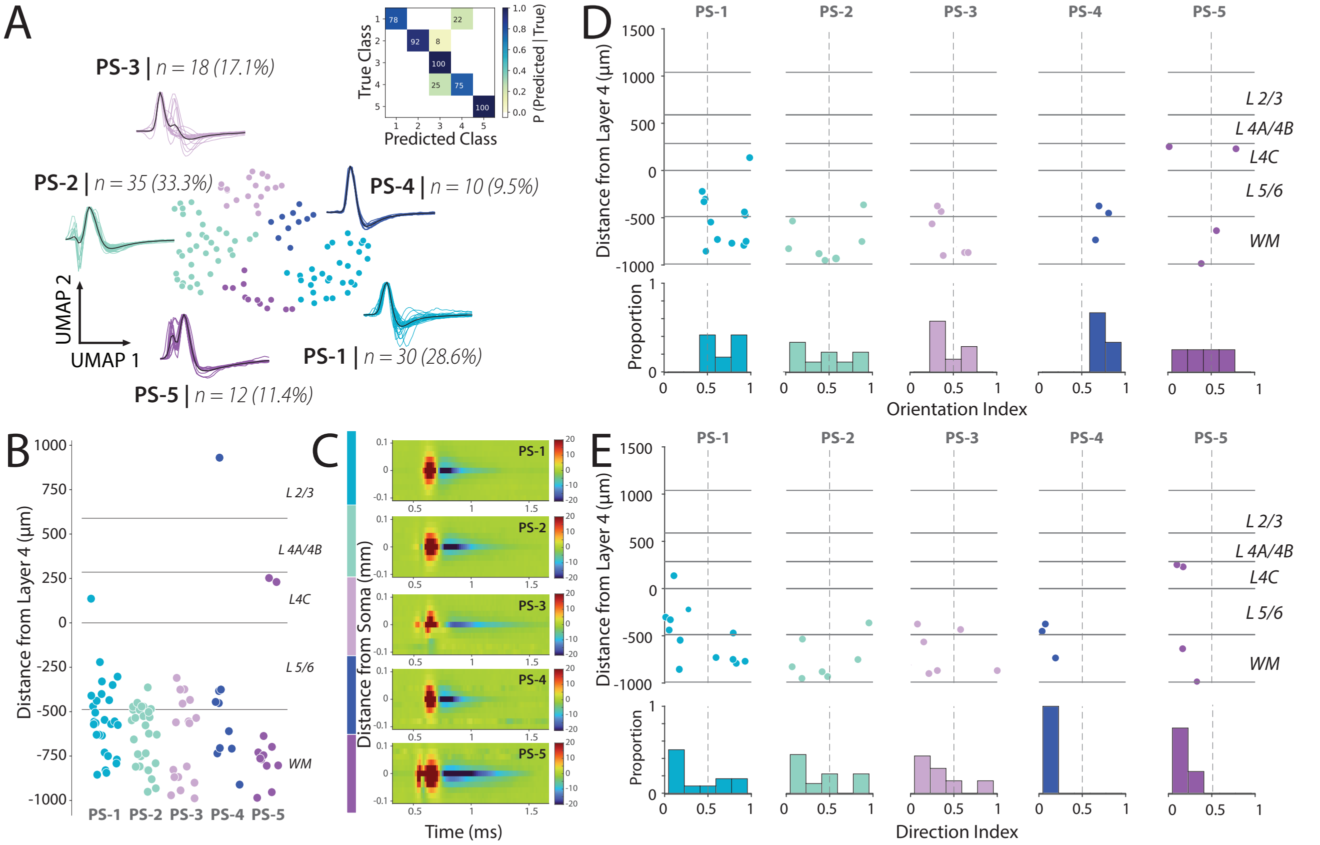

Figure S7: **Positive waveform clusters are largely in white matter and show a diversity of visual responses** (**A**) Scatter plot of WaveMAP clustering on 104 positive spiking waveforms (N_neighbors = 20; MIN_DIST = 0.2; RESOLUTION = 1.0) *Inset*, Confusion Matrix showing gradient boosted decision tree classifier with five-fold cross-validation. The main diagonal shows accuracy of waveform classification for each cluster, and off diagonals show misclassification percentages. (**B**) Laminar distribution of 5 positive spiking (PS) clusters across average laminar boundaries. Points are randomly jittered on the x-axis. (**C**) Average multichannel extracellular waveforms per cluster. Cluster color is shown on the left, and the colorbar shows the scale standardized for all clusters to map linearly between -20 and 20 μv. (**D**) Of the 105 positive spiking units, 32 were responsive to visual stimuli. *Top*, Scatter plot of orientation index and scaled laminar depth separated by cluster for positive spiking units. *Bottom*, Panel below the scatter plots shows histograms of the orientation index. The vertical dashed center lines show the boundary between not selective (left of center) and selective (right of center). While the numbers are too small to strong statistical conclusions, clusters PS-1 and PS-4 have strong orientation selectivity but low direction selectivity similar to TP-1 type B neurons (Fig. S6B). (**E**) *Top*, Scatter plot of direction index and scaled laminar depth separated by cluster for positive spiking units. *Bottom*, Panel below the scatter plots shows histograms of the direction index. The vertical dashed center lines show the boundary between not selective (left of center) and selective (right of center). In general, clusters PS1-5 show modest direction selectivity.

Table S1: Distribution of Neuronal Clusters Across Cortical Layers

Layer	Narrow-Spiking (NS)										Tri-phasic (TP)		Broad-Spiking (BS)									
	NS-1		NS-2		NS-3		NS-4		NS Total				BS-1		BS-2		BS-3		BS-4		BS Total	
	n	%	n	%	n	%	n	%	n	%	n	%	n	%	n	%	n	%	n	%	n	%
2/3	6	15.4	4	6.3	5	9.6	5	11.4	20	10.1	3	5.1	29	24.6	14	21.5	28	29.2	12	17.4	83	23.9
4AB	19	48.7	28	43.8	25	48.1	23	52.3	95	47.7	12	20.3	24	20.3	10	15.4	13	13.5	8	11.6	55	15.8
4C	13	33.3	29	45.3	10	19.2	7	15.9	59	29.6	19	32.2	52	44.1	36	55.4	40	41.7	9	13.0	137	39.4
5/6	1	2.6	3	4.7	12	23.1	9	20.5	25	12.6	15	25.4	13	11.0	5	7.7	15	15.6	35	50.7	68	19.5
WM	0	0.0	0	0.0	0	0.0	0	0.0	0	0.0	10	16.9	0	0.0	0	0.0	0	0.0	5	7.2	5	1.4
Total	39		64		52		44		199		59		118		65		96		69		348	

bioRxiv preprint doi: https://doi.org/10.1101/2025.05.14.653875; this version posted June 25, 2025. The copyright holder for this preprint (which was not certified by peer review) is the author/funder, who has granted bioRxiv a license to display the preprint in perpetuity. It is made available under aCC-BY 4.0 International license.
